# Database of butterfly and moth observations in the Netherlands: research from 1947-2020

**DOI:** 10.3897/BDJ.10.e78784

**Published:** 2022-04-07

**Authors:** François J. A. Slieker, Niels Raes

**Affiliations:** 1 Natural History Museum Rotterdam, Rotterdam, Netherlands Natural History Museum Rotterdam Rotterdam Netherlands; 2 Netherlands Biodiversity Information Facility (NLBIF) - Naturalis Biodiversity Center, Leiden, Netherlands Netherlands Biodiversity Information Facility (NLBIF) - Naturalis Biodiversity Center Leiden Netherlands

**Keywords:** Lepidoptera, butterflies, moths, Netherlands, observations, occurrence, database

## Abstract

**Background:**

The Natural History Museum Rotterdam (NMR) is a regional natural history museum in The Netherlands that focuses on nature and biodiversity of the city of Rotterdam and its surroundings. Bureau Stadsnatuur Rotterdam (bSR) is part of the NMR and collects, mainly on behalf of third parties, data on the flora and fauna from primarily urban areas. The NMR has received a large amount of observation data (1,363 different species in 886,902 observations), in particular of moths and mainly from the Provinces of Zuid-Holland, Noord-Holland and Noord-Brabant from the period 1947-2020. The observation dataset was compiled and standardised from 18 different datasets and stored in a database and published at the Global Biodiversity Information Facility (GBIF).

**New information:**

For the first time, a large butterfly and moth observations dataset with historical distribution data for The Netherlands is mobilised and serves as a baseline lepidopteran biodiversity record.

## Introduction

Lepidopterans are an important group of arthropods that are particularly sensitive to global climate change and relatively fast change of environmental factors. Climate change will shift geographical ranges of species and will have significant effect on dispersal capacity. Changes in climate conditions have affected survival, reproduction and food availability (Kocsis and Hufnagel 2011).

In The Netherlands, butterflies and moths are under great pressure, both in numbers and in species diversity. Since 1940, one quarter of Dutch butterfly species has disappeared from our country and over 40% of the remaining species are threatened (Bos et al. 2006). Dutch moth data from 1980 onwards show a disturbing picture with a sharp decline in number of individuals of most species, as well as the disappearance of species from the Dutch fauna (Ellis et al. 2013). At present, data on Dutch lepidopterans prior to 1980 are of limited availability and make it difficult to interpret current changes (Bos et al. 2006, Ellis et al. 2013). Historical observations are, therefore, of crucial importance to gain a better understanding in the development and trends of the Dutch butterfly fauna. The database contains 1,363 different species in 886,902 observations (Slieker 2021) and was compiled from 18 different datasets (Table 1), some presented in spreadsheet format, some on handwritten lists, coverering the time period from 1947-2013.

## Sampling methods

### Study extent

The Netherlands, Provinces of Zuid-Holland, Noord-Holland, Noord-Brabant, Limburg, Utrecht and Gelderland.

### Sampling description

Each occurrence contained fundamental information, such as location, coordinates, date, name of observer and identifier. Coordinates were determined directly on site with the help of a GPS device (554,476 occurrences). In other cases, Google Maps (2020) and Google Earth were used. The margin of error in both measurements of coordinates is 11 m. The accuracy of determining coordinates is up to the fourth digit. In all occurrences, the WGS-84 geodetic datum is used.

One subset of data (135,596 occurrences) contains environmental data in the “dynamicProperties” field https://www.gbif.org/occurrence/search?dataset_key=6db2a74e-98c5-4be3-ae30-3ec8dc68b0f4&year=1964. Measured values include cloud cover, mean sea level, pressure (HPa), relative humidity percentage, lunar surface illumination percentage, rainfall (mm), air temperature (°C), mean wind direction, windforce (Beaufort) and windspeed (m/s).

### Quality control

Voucher specimens were taken from two subsets of data (Table 1, partial datasets 01 and 02) and obtained by sampling with a modified Robinson moth trap. These specimens are kept in the Rotterdam Museum of Natural History. Observations in partial datasets 03 t/m 17 (Table 1) were made by experienced entomologists, mutually verifying each other's observations. Partial dataset 18 contains data monitored by a team of senior urban ecologists, including experienced entomologists.

## Geographic coverage

### Description

**Figure 2.**
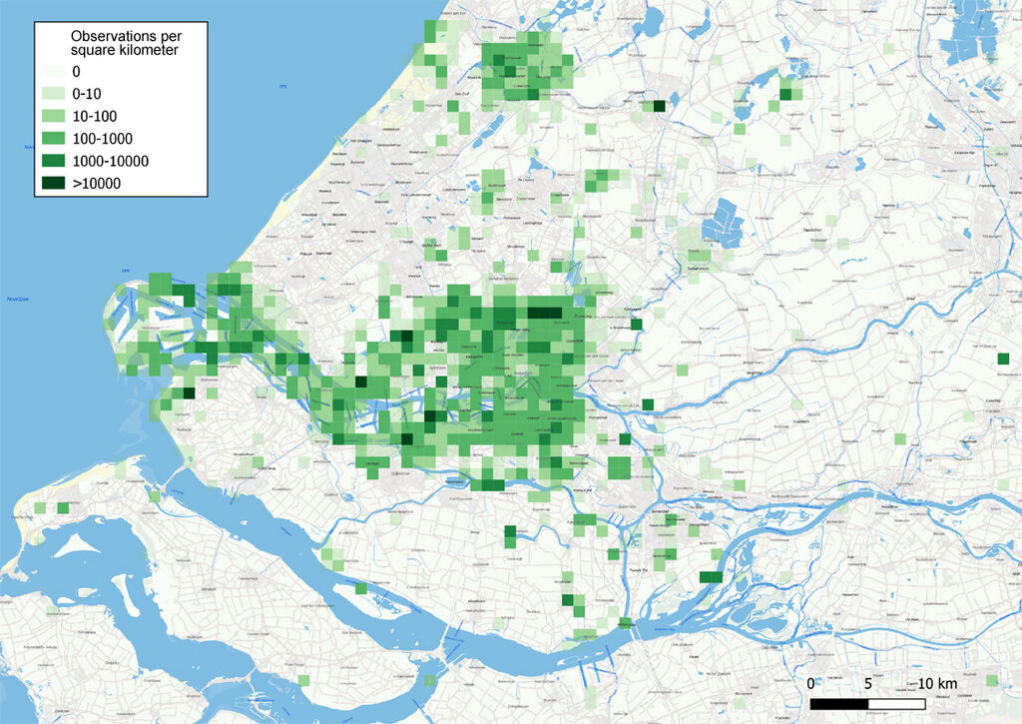
Overview of observations made in the Dutch Province of Zuid-Holland (91.6% of all sightings in the total dataset).

The dataset contains information about the occurrence of Lepidopterans in The Netherlands, primarily originating from six Dutch Provinces: Noord-Holland, Zuid-Holland, Noord-Brabant, Limburg, Utrecht and Gelderland. The different sites cover a wide variety of habitats ranging from dune areas with shrubs, low dune grassland, foreland osiers, urban parks, as well as new land reclaimed from the sea. A density map with the records is shown in Fig. 2.

### Coordinates

 and 52.2066 - 52.9352 N Latitude; and 3.4494 - 7.1188 E Longitude.

## Taxonomic coverage

### Taxa included

**Table:** 

Rank	Scientific Name	Common Name
kingdom	Animalia	Animals
phylum	Arthropoda	Arthropods
class	Insecta	Insects
order	Lepidoptera	lepidopterans
family	Adelidae	
family	Alucitidae	
family	Argyresthiidae	
family	Autostichidae	
family	Batrachedridae	
family	Bedelliidae	
family	Blastobasidae	
family	Bucculatricidae	
family	Choreutidae	
family	Coleophoridae	
family	Cosmopterigidae	
family	Cossidae	
family	Crambidae	
family	Depressariidae	
family	Drepanidae	
family	Elachistidae	
family	Epermeniidae	
family	Erebidae	
family	Eriocraniidae	
family	Ethmiidae	
family	Gelechiidae	
family	Geometridae	
family	Glyphipterigidae	
family	Gracillariidae	
family	Heliozelidae	
family	Hepialidae	
family	Hesperiidae	
family	Incurvariidae	
family	Lasiocampidae	
family	Limacodidae	
family	Lycaenidae	
family	Lyonetiidae	
family	Micropterigidae	
family	Momphidae	
family	Nepticulidae	
family	Noctuidae	
family	Nolidae	
family	Notodontidae	
family	Nymphalidae	
family	Oecophoridae	
family	Pantheidae	
family	Papilionidae	
family	Pieridae	
family	Plutellidae	
family	Psychidae	
family	Pterophoridae	
family	Pyralidae	
family	Saturniidae	
family	Sesiidae	
family	Sphingidae	
family	Stathmopodidae	
family	Thyrididae	
family	Tineidae	
family	Tischeriidae	
family	Tortricidae	
family	Yponomeutidae	
family	Ypsolophidae	
family	Zygaenidae	

## Temporal coverage

**Data range:** 1947-1-01 – 2020-12-31.

## Usage licence

### Usage licence

Creative Commons Public Domain Waiver (CC-Zero)

## Data resources

### Data package title

Natural History Museum Rotterdam - Observations

### Resource link

https://www.gbif.org/dataset/6db2a74e-98c5-4be3-ae30-3ec8dc68b0f4

### Number of data sets

1

### Data set 1.

#### Data set name

Natural History Museum Rotterdam - Observations

#### Data format

DwC & GBIF API terms

#### Number of columns

58

#### Download URL

https://doi.org/10.15468/czfn9y

#### Data format version

DwC 2021-07-15 & GBIF API v.1

#### Description

A set of 886,902 observations of Lepidoptera made between 1947 and 2020 in The Netherlands compiled from 18 datasets (Table 1), partially including phenological data.

**Data set 1.** 

Column label	Column description
catalogNumber	An identifier (preferably unique) for the record within the dataset or collection.
recordNumber	An identifier given to the Occurrence at the time it was recorded. Often serves as a link between field notes and an Occurrence record, such as a specimen collector's number.
occurrenceID	A single globally unique identifier for the occurrence record as provided by the publisher.
kingdom	The full scientific name of the kingdom in which the taxon is classified.
phylum	The full scientific name of the phylum or division in which the taxon is classified.
class	The full scientific name of the class in which the taxon is classified.
order	The full scientific name of the order in which the taxon is classified.
family	The full scientific name of the family in which the taxon is classified.
genus	The full scientific name of the genus in which the taxon is classified.
specificEpithet	The name of the first or species epithet of the scientificName.
infraspecificEpithet	The name of the lowest or terminal infraspecific epithet of the scientificName, excluding any rank designation.
taxonRank	The taxonomic rank of the most specific name in the scientificName.
scientificName	The full scientific name, with authorship and date information if known.
identifiedBy	A list (concatenated and separated) of names of people, groups or organisations who assigned the Taxon to the subject.
countryCode	The standard code for the country in which the Location occurs.
locality	The specific description of the place.
stateProvince	The name of the next smaller administrative region than country (state, province, canton, department, region etc.) in which the Location occurs.
occurrenceStatus	A statement about the presence or absence of a Taxon at a Location.
individualCount	The number of individuals present at the time of the Occurrence.
collectionID	An identifier for the collection or dataset from which the record was derived.
decimalLatitude	Latitude in decimals between -90 and 90, based on WGS 84.
decimalLongitude	Longitude in decimals between -180 and 180, based on WGS 84.
coordinateUncertaintyInMetres	The horizontal distance (in metres) from the given decimalLatitude and decimalLongitude describing the smallest circle containing the whole of the Location.
coordinatePrecision	A decimal representation of the precision of the coordinates given in the decimalLatitude and decimalLongitude.
minimumElevationInMetres	The lower limit of the range of elevation (altitude, usually above sea level), in metres.
rightsHolder	A person or organisation owning or managing rights over the resource.
recordedBy	A list (concatenated and separated) of names of people, groups or organisations responsible for recording the original Occurrence. The primary collector or observer, especially one who applies a personal identifier (recordNumber), should be listed first.
establishmentMeans	Statement about whether an organism or organisms have been introduced to a given place and time through the direct or indirect activity of modern humans.
eventDate	The date-time or interval during which an Event occurred.
day	The integer day of the month on which the Event occurred.
month	The integer month in which the Event occurred.
year	The four-digit year in which the Event occurred, according to the Common Era Calendar.
bibliographicCitation	A bibliographic reference for the resource as a statement indicating how this record should be cited (attributed) when used.
dynamicProperties	A list of additional measurements, facts, characteristics, or assertions about the record. Meant to provide a mechanism for structured content.
basisOfRecord	The specific nature of the data record; for example, PreservedSpecimen, FossilSpecimen, LivingSpecimen, MaterialSample, Event, HumanObservation, MachineObservation, Taxon, Occurrence, MaterialCitation.
institutionCode	The name (or acronym) in use by the institution having custody of the object(s) or information referred to in the record.
collectionCode	The name, acronym, coden or initialism identifying the collection or dataset from which the record was derived.
continent	The name of the continent in which the Location occurs.
country	The name of the country or major administrative unit in which the Location occurs.
eventID	An identifier for the set of information associated with an Event (something that occurs at a place and time). May be a global unique identifier or an identifier specific to the dataset.
geodeticDatum	The ellipsoid, geodetic datum or spatial reference system (SRS) upon which the geographic coordinates given in decimalLatitude and decimalLongitude are based.
georeferencedBy	A list (concatenated and separated) of names of people, groups, or organisations who determined the georeference (spatial representation) for the Location.
georeferenceVerificationStatus	A categorical description of the extent to which the georeference has been verified to represent the best possible spatial description for the Location of the Occurrence.
habitat	A category or description of the habitat in which the Event occurred.
institutionID	An identifier for the institution having custody of the object(s) or information referred to in the record.
language	A language of the resource.
lifeStage	The age class or life stage of the Organism(s) at the time the Occurrence was recorded.
nomenclaturalCode	The nomenclatural code (or codes in the case of an ambiregnal name) under which the scientificName is constructed.
occurrenceRemarks	Comments or notes about the Occurrence.
organismQuantity	A number or enumeration value for the quantity of organisms.
organismQuantityType	The type of quantification system used for the quantity of organisms.
samplingProtocol	The names of, references to, or descriptions of the methods or protocols used during an Event.
taxonomicStatus	The status of the use of the scientificName as a label for a taxon. Requires taxonomic opinion to define the scope of a taxon. Rules of priority then are used to define the taxonomic status of the nomenclature contained in that scope, combined with the experts' opinion. It must be linked to a specific taxonomic reference that defines the concept.
type	The nature or genre of the resource.
verbatimCoordinates	The verbatim original spatial coordinates of the Location. The coordinate ellipsoid, geodeticDatum or full Spatial Reference System (SRS) for these coordinates should be stored in verbatimSRS and the coordinate system should be stored in verbatimCoordinateSystem.
verbatimCoordinateSystem	The coordinate format for the verbatimLatitude and verbatimLongitude or the verbatimCoordinates of the Location.
vernacularName	A common or vernacular name.
license	A legal document giving official permission to do something with the resource.

## Figures and Tables

**Figure 1.**
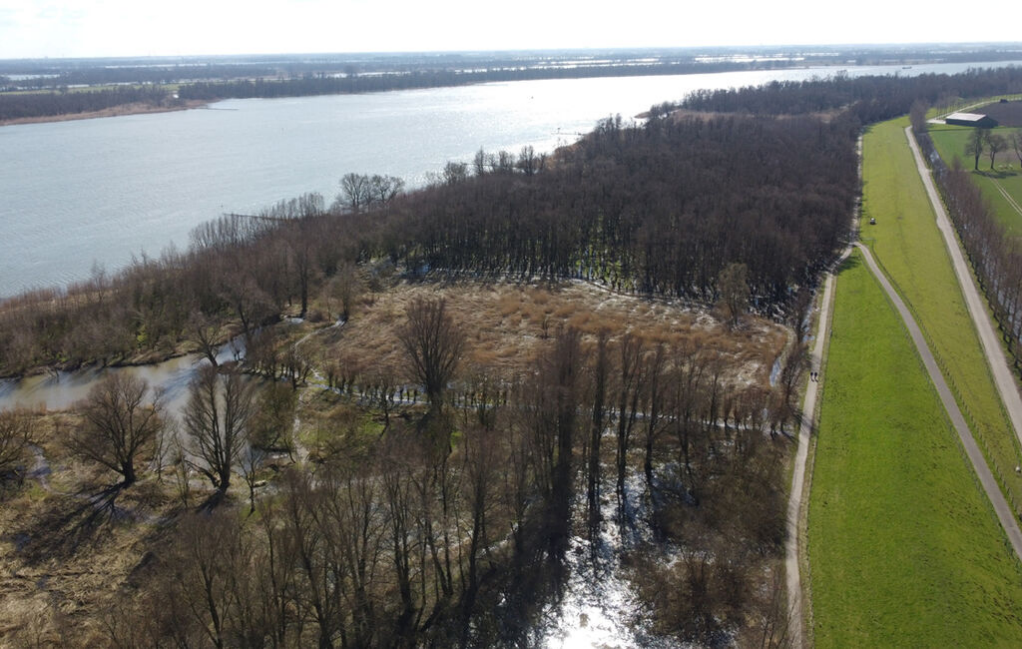
Netherlands, Dordrecht, Kop van 't Land, observation site of moths listed in dataset 06. Aerial image courtesy of Manuel Schiesser.

**Table 1.** Overview of the 18 datasets used to compile the dataset.

Partial dataset	Locality	Period	Observations	Observer(s)
01	Netherlands, Zuid-Holland, Westvoorne, Oostvoorne, Weeversduin	1964	135,329	R.Vis, D.A. Vestergaard and and J.G. v.d. Made (Vestergaard et al. 1973)
02	Netherlands, Zuid-Holland, Westvoorne, Oostvoorne, Weeversduin	1963	130,327	R.Vis, D.A. Vestergaard and and J.G. v.d. Made (Vestergaard et al. 1973)
03	Netherlands, Zuid-Holland, Wassenaar, Bierlap	1955-1956	4,716	J.A.W. Lucas, and A. van Tol
04	Netherlands, Zuid-Holland, Rotterdam, Hoek van Holland	1999-2005	7,375	N.W. Elfferich, J.A.W. Lucas, and R. van Tilborg
05	Netherlands, Zuid-Holland, Albrandswaard, Rhoon	1995-1999	7,369	N.W. Elfferich, J.A.W. Lucas, and R. van Tilborg
06	Netherlands, Zuid-Holland, Dordrecht, Kop van ’t Land (Fig. 1)	1987-2005	8,727	R. Vis, and D.O. Visser (Vis and Visser 2007)
07	Netherlands, Zuid-Holland, Krimpenerwaard, Lekkerkerk, Bakkerswaal	1985-1988	9,590	N.W. Elfferich, and J.A.W. Lucas
08	Netherlands, Zuid-Holland, Westland, ’s-Gravenzande, Staelduinse Bos	1989-1993	7,441	N.W. Elfferich, and J.A.W. Lucas
09	Netherlands, Zuid-Holland, Zuidplas, Nieuwerkerk a/d IJssel, Hitland	2000-2006	2,597	N.W. Elfferich, J.A.W. Lucas, R. van Tilborg
10	Netherlands, Noord-Brabant, Rucphen, Schijf	2000	912	Elfferich, J.A.W. Lucas, and R. van Tilborg
11	Netherlands, Zuid-Holland, Westland, ’s-Gravenzande, Staelduinse Bos	1989-1993	1,525	A.M. van Oosten, and N.W. Elfferich
12	Netherlands, butterflies from multiple localities	1947-2010	909	R. Vis
13	Netherlands, Noord-Brabant, Boxtel, Kampinasche Heide	1959	2,148	R. Vis, D.A. Vestergaard, and J.G. van der Made
14	Netherlands, Overijssel, Dinkelland, Denekamp	2003	627	R. Vis, and D.O. Visser
15	Netherlands, multiple localities	1957-2013	8,816	R. Vis
16	Netherlands, multiple localities	1959, 2008-2009	1,043	R. Vis, D.O. Visser, J.G. van der Made
17	Netherlands, Noord-Holland, Texel, Hoge Berg	2010	190	J. Kuchlein
18	Netherlands, multiple localities	1992-2020	557,261	Team urban ecologists from Bureau Stadsnatuur Rotterdam
